# A wearable gait lab powered by sensor-driven digital twins for quantitative biomechanical analysis post-stroke

**DOI:** 10.1017/wtc.2024.14

**Published:** 2024-11-14

**Authors:** Donatella Simonetti, Maartje Hendriks, Bart Koopman, Noel Keijsers, Massimo Sartori

**Affiliations:** 1 Biomechanical Engineering Department, University of Twente, 7522 NB Enschede, Netherlands; 2 Sint MaartensKlinik, 6574 NA Ubbergen, Netherlands

**Keywords:** wearable, gait, kinematics, kinetics, musculoskeletal model

## Abstract

Commonly, quantitative gait analysis post-stroke is performed in fully equipped laboratories housing costly technologies for quantitative evaluation of a patient’s movement capacity. Combining such technologies with an electromyography (EMG)-driven musculoskeletal model can estimate muscle force properties non-invasively, offering clinicians insights into motor impairment mechanisms. However, lab-constrained areas and time-demanding sensor setup and data processing limit the practicality of these technologies in routine clinical care. We presented wearable technology featuring a multi-channel EMG-sensorized garment and an automated muscle localization technique. This allows unsupervised computation of muscle-specific activations, combined with five inertial measurement units (IMUs) for assessing joint kinematics and kinetics during various walking speeds. Finally, the wearable system was combined with a person-specific EMG-driven musculoskeletal model (referred to as human digital twins), enabling the quantitative assessment of movement capacity at a muscle-tendon level. This human digital twin facilitates the estimation of ankle dorsi-plantar flexion torque resulting from individual muscle-tendon forces. Results demonstrate the wearable technology’s capability to extract joint kinematics and kinetics. When combined with EMG signals to drive a musculoskeletal model, it yields reasonable estimates of ankle dorsi-plantar flexion torques (*R*^2^ = 0.65 ± 0.21) across different walking speeds for post-stroke individuals. Notably, EMG signals revealing an individual’s control strategy compensate for inaccuracies in IMU-derived kinetics and kinematics when input into a musculoskeletal model. Our proposed wearable technology holds promise for estimating muscle kinetics and resulting joint torque in time-limited and space-constrained environments. It represents a crucial step toward translating human movement biomechanics outside of controlled lab environments for effective motor impairment monitoring.

## 1. Introduction

Gait assessment is commonly used to evaluate the movement capacity of a person with motor disorders such as those resulting from conditions like stroke (Mohan et al., 2021), spinal cord injury (Barbeau et al., 1999), and cerebral palsy (Dickens & Smith, 2006). In the last two decades, fully equipped biomechanics laboratories with camera-based motion tracking systems, stationary force plates, and electromyography (EMG) sensors have played a pivotal role in enabling quantitative gait analysis (Nadeau et al., 2013; Klöpfer-Krämer et al., 2020).

By using motion capture technologies, force sensors, and EMG, we can record human movements, ground reaction forces (GRFs), and muscle-specific activation patterns, respectively. Data on body motion and external forces can be combined with a multi-body dynamic model to simulate the motions, e.g., joint kinematics and joint moments (Winter, 2009). This methodology alone cannot yield insights into the force-generating properties of the underlying muscles. By estimating musculoskeletal properties such as muscle activations and muscle forces, clinicians can get a better understanding of the underlying causes of a person’s movement impairments, develop personalized rehabilitation interventions, and monitor the progress for optimal recovery. Since the musculoskeletal system is redundant, i.e., many muscles act on the same joint, and innumerable solutions are possible to obtain the same movement, knowing inverse dynamics (ID)-derived moments is not enough to estimate the force contribution of each muscle acting on the same joint.

A person-specific musculoskeletal model can facilitate the non-invasive estimation of muscle forces. By employing measured body kinetic and kinematic data and by minimizing an objective function describing pre-defined physiological criteria (Pedotti et al., 1978; Crowninshield & Brand, 1981; Davy & Audu, 1987), muscle forces and muscle activations can be computed. However, since the computation of muscle forces is based on optimization techniques and not on muscle-related measured data, its applicability to neurologically impaired individuals is suboptimal (Simpson et al., 2015). The estimation of muscle activation and muscle forces starting from positions and external forces can be challenged by abnormal neuromuscular patterns, underlying co-contraction, spasticity, or the presence of silent muscles. However, measured muscle activation input in a person-specific musculoskeletal model (referred to as EMG-driven musculoskeletal model) can better detect these abnormalities. EMG-driven models provide a digital representation of a person’s musculoskeletal anatomy, where individual muscle-tendon units (MTUs) are driven by measured EMGs. This digital representation is referred to as a human digital twin. Human digital twins bridge the physical and digital worlds, enabling monitoring, simulation, and analysis of a person’s movement capacity at a muscle-tendon level under different conditions. In a clinical context, the use of human digital twins can facilitate the identification of the causes behind a movement impairment and help optimize rehabilitation treatments. In the last decades, EMG-driven musculoskeletal models have enabled the non-invasive estimation of muscle-tendon forces and resulting joint torques in healthy (Sartori et al., 2012) and neurologically impaired individuals (Knarr et al., 2014; Manal et al., 2012). The presence of such parameters can describe how the muscles are coordinated during a specific movement and hence help gain a thorough understanding of the underlying mechanisms provoking the motor impairment. However, the implementation of EMG-driven models has been primarily restricted to fully equipped laboratories (Sartori et al., 2012; Durandau et al., 2018). The high costs of cameras and force plates, the lab-constrained measurement area, as well as the time-demanding sensor setup and processing, have limited the applicability of this technology to tackle daily life problems in healthcare environments with a high demand for gait assessment.

There is a need to replace conventional laboratory systems with a fully wearable and portable gait assessment tool. In the present study, we proposed a wearable technology comprising a multi-channel EMG-sensorized garment and an automated muscle localization algorithm combined with five inertial measurement units (IMU) as well as an EMG-driven musculoskeletal model. This can allow the computation of (1) knee and ankle angles and ID-derived ankle dorsi-plantar flexion torque, and (2) ankle dorsi-plantar flexion torque estimated using an EMG-driven musculoskeletal model during walking at different speeds in post-stroke individuals. With the proposed solution, we investigated the possibility of (1) removing the need for laboratory-based technologies, such as force plates and camera-based tracking systems, by using five IMU sensors, and (2) using IMU data and a person-specific musculoskeletal model to estimate knee and ankle angles, right and left GRFs, and ID-derived ankle dorsi-plantar flexion torques. Furthermore, we hypothesized that (3) we could calibrate a person-specific EMG-driven musculoskeletal model and estimate ankle dorsi-plantar flexion torque using joint angles and ankle dorsi-plantar flexion torque derived from five IMUs in conjunction with muscle activations derived from the garment-embedded EMG signals. We hypothesized that the IMU and EMG-driven ankle dorsi-plantar flexion torque could be estimated with comparable accuracy (*R*^2^ > 0.7 and RMSE < 0.3 Nm/kg) to the ankle dorsi-plantar flexion torque retrieved via an inverse dynamics approach informed by joint kinematics and kinetics retrieved via lab-bound technologies (multi-camera tracking systems and in-ground force plates).

We focused on the ankle joint since typical gait retraining in post-stroke survivors has focused on the impairment of plantar flexor muscles that are the primary contributors to forward propulsion and the swing initiation phases (Embrey et al., 2010; Kesar et al., 2009). Previous studies suggested that a rehabilitation strategy aiming at increasing paretic forward propulsion and swing initiation in a post-stroke population can help improve gait performance (Peterson et al., 2010).

Wearable sensors have been widely used to compute joint torque via ID. Previous studies combined IMUs with other portable technologies (portable force plates or pressure insoles) (Liu et al., 2014; Khurelbaatar et al., 2015; Wang et al., 2022) or machine learning algorithms (Li et al., 2016; Stetter et al., 2020), to compute lower limb joint moments. Other studies have integrated IMUs with person-specific biomechanical models to compute knee and ankle moments during walking (Fukutoku et al., 2020), as well as hip moments during balance tasks (Noamani et al., 2020). However, all these works applied wearable sensors to healthy participants.

Similarly, EMG-driven musculoskeletal models have been used to estimate joint moments based on underlying muscle forces. Several studies have used laboratory-informed signals together with a person-specific musculoskeletal model to estimate joint moments in healthy (Sartori et al., 2012; Durandau et al., 2018; Tagliapietra et al., 2015) and neurologically impaired individuals (Knarr et al., 2014; Manal et al., 2012). Furthermore, EMG-driven models together with IMUs (Han et al., 2015) or machine learning techniques (Wu et al., 2021; Heine et al., 2011) have been employed to estimate joint moments of the upper and lower limb, albeit typically for healthy individuals. Our previous studies combined an EMG-sensorized leg garment, automated muscle localization techniques, and an EMG-driven model to estimate ankle dorsi-plantar flexion torque (Simonetti et al., 2023) during walking in post-stroke and healthy participants. However, the acquisition of joint kinematics and kinetics relied still on lab-bound technologies such as 16-camera tracking systems and two floor-embedded force plates. The use of these non-wearable and expensive laboratory setups prevents the application of our EMG-sensorized leg garments and EMG-driven models to out-of-lab scenarios. Moreover, the lengthy setup (e.g., placement of multiple markers on the body for joint kinematics processing) is not compatible with the limited time available per patient in clinical settings. Our current research tackles these limitations by introducing a fully wearable technology that uses five IMUs to estimate both joint kinematics and foot-ground reaction forces needed for the EMG-driven model calibration. This can potentially facilitate the transfer of these technologies into clinical settings. To the best of the authors’ knowledge, no previous studies combine the estimation of muscle kinetics and resulting joint torque by integrating EMG-sensorized garments, an automated muscle localization algorithm, wireless and wearable IMU sensors, and an EMG-driven musculoskeletal model with validation on healthy and post-stroke individuals.

In the subsequent sections, we elaborate on the experimental procedures along with the data processing for the laboratory-based and fully wearable systems. We showed the effectiveness of IMU linear accelerations and orientations in computing joint kinetics and kinematics, respectively, compared to a standard laboratory system (Section 3). Furthermore, we demonstrated that EMG signals revealing the person-specific control strategy largely impact the final torque estimation and compensate for IMU-derived joint angle inaccuracies when input to a musculoskeletal model (Section 3). We applied the same methodology to a control group comprising nine healthy participants and validated the IMU-based estimation of joint kinetics and kinematics, as well as IMU-derived joint angles and bipolar EMG-driven estimation of ankle moments (Supplementary Material). Finally, we discuss the results, including study limitations and future work (Section 4).

## 2. Methods

### 2.1. Experimental procedures

Four males with hemiparetic post-stroke (age = 53.8 ± 8.0 years, height = 177.5 ± 4.3 cm, weight = 94.2 ± 20.1 kg) (Table 1) were recruited for participation through the Sint Maartenskliniek (Nijmegen, The Netherlands). The study (reference number 2022–13658) was approved by the regional medical ethics committee of Eastern Netherlands (METC Oost-Nederland). Each participant completed three tasks: a static standing pose in a neutral position (10 s), walking at a self-selected comfortable speed, and walking as fast as possible. A minimum of 10 gait cycles in each walking condition were recorded.

**Table 1.** Post-stroke participants information

	Age	Weight (kg)	Height (m)	Time since stroke (months)	Stroke type	FAC score (0–5)	Affected side
p01	44	78	1.72	8	ischemic	5	left
p02	57	128	1.83	36	hemorragic	5	left
p03	49	80.2	1.75	3	ischemic	5	right
p04	65	90.4	1.80	4	hemorragic	5	left

*Abbreviation:* FAC = Functional Ambulation Category.

**Figure 1.**
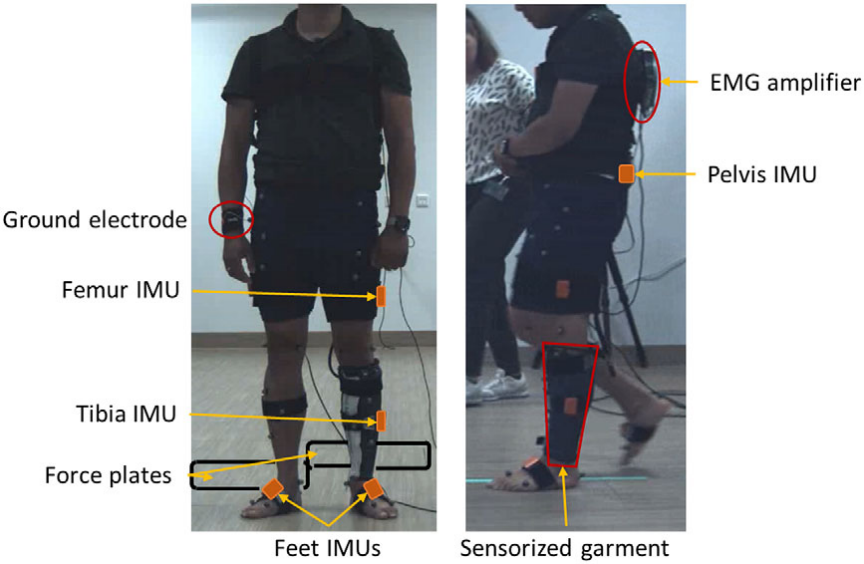
Experimental setup.

EMG data were recorded using an EMG-sensorized garment (Figure 1) embedded with a grid of 64 EMG electrodes equally distributed and surrounding the leg of the affected side. Prior to donning the garment, the skin was moistened with salty water to reduce the electrodes’ impedance. A ground electrode was attached using a wet wristband. All EMG channels were connected to a multi-channel amplifier (SAGA 64+, Data Recorder, TMSi, Odendzaal, The Netherlands) worn on the back of the participant and connected to a desktop station (SAGA 64+, Docking Station, TMSi, Odendzaal, The Netherlands) through an optical fiber cable. The EMG signals were recorded at a sampling rate of 2000 Hz and amplified against the average of all 64 connected floating inputs, i.e., average reference mode, with the exception of the ground electrode. A detailed description of the EMG-sensorized garment is presented in our previous work (Simonetti et al., 2023).

Kinematics data were captured using five IMUs (XsensTM MTw, Movella, Enschede, The Netherlands) and a 3D motion tracking system (Vicon, New York (NY), USA). IMUs were attached with velcro straps: on the sacrum at the midway point between the line connecting the left and right posterior superior iliac spine, on each foot on the midfoot region, and on the impaired leg on the thigh on the mid-femur lateral region, and on the tibia below the tibial tuberosity (Figure 1). The MT Manager software (MT manager 2019.2, Movella, Enschede, The Netherlands) was used for wireless data recording from the IMUs, with a sampling rate of 100 Hz. Thirty-seven reflective markers were placed on bony landmarks and leg segments, as previously described (Sartori et al., 2012). Data were acquired at 100 Hz using 16 optical cameras (eight Vicon Bonita cameras, four Vicon Vero 2.2 cameras, and four Vicon Vue video cameras). Simultaneously, 3D GRFs were recorded at 1000 Hz via two floor-embedded force plates (Kistler 9286BA, Kistler, Winterthur, Switzerland). The EMG and kinematics tracking systems were synchronized by using the digital input of the Data Recorder (DIGI) to record the motion tracking system’s start and end events.

### 2.2. Data processing

Signal processing of the raw EMG, kinetic, and kinematic data was done on Matlab (Matlab2020a, MathWorks, Natick (MA), USA).

#### 2.2.1. Kinematic and kinetic data

The kinematic and kinetic data from the optical motion tracking system and the force plates were low-pass filtered at 6 Hz with a zero-lag 2^nd^ order Butterworth filter. Linear and angular accelerations and velocities extracted from the IMU software were low-pass filtered at 3 Hz with a zero-lag 2^nd^ order Butterworth filter. A lower cut-off frequency with respect to the kinetic and kinematic data obtained from lab systems was chosen to reduce the noise introduced by relative movement between sensors and the underlying segment.

#### 2.2.2. EMG data

For the post-stroke individuals, the raw EMG signals were amplified with a gain of 23 and automatically inspected to identify noisy channels, that is, channels with large voltage fluctuations due to movement artifacts as described (Simonetti et al., 2023). All noisy channels were set to zero. The raw EMG signals measured from the remaining channels went through re-referencing processing (see detailed processing in (Simonetti et al., 2023)) to remove the noise introduced by the noisy channels during the average reference amplification modality. The rereferenced EMG signals were processed to extract linear envelopes. First, the re-referenced EMG signals were high-pass filtered at 20 Hz using a zero-lag 2^nd^ order Butterworth filter and fully rectified. Afterward, the rectified EMG signals went through a moving median filter with a moving window length of 0.16 s to obtain an equivalent behavior to a low-pass filter with a 6 Hz cut-off frequency (Conforto et al., 1999). The moving median filter allowed removing the remaining spikes due to movement artifacts. The resulting linear envelopes of each channel were normalized against the maximum linear envelopes’ value extracted among all performed tasks.

The multi-channel EMG clustering developed in (Simonetti et al., 2023) was applied to the 64 normalized linear envelopes from three gait cycles at a comfortable walking speed to extract muscle-specific activations for the following seven muscles: tibialis anterior, extensor hallucis longus, medial and lateral gastrocnemius, soleus, peroneus brevis, and longus.

### 2.3. Wearable lab

#### 2.3.1. Scaling and Inverse kinematics

**Figure 2.**
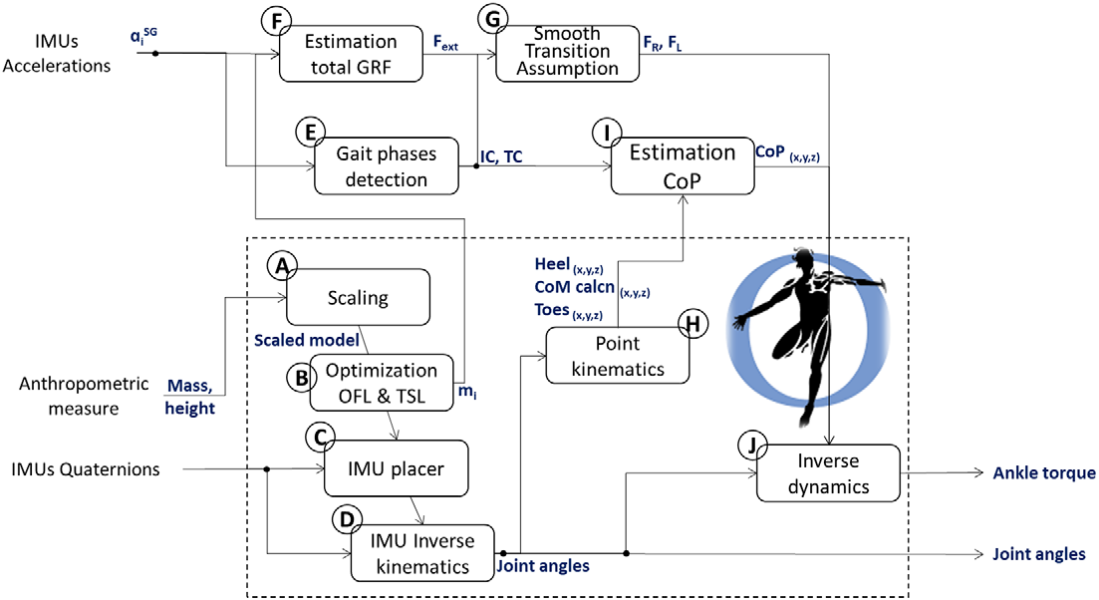
Schematics of the IMU-based pipeline to extract joint angles and ankle torques from accelerations and quaternions as well as person-specific anthropometric measures. Using the OpenSim software, the mass and height are used to scale the default musculoskeletal model (a) to the specific participant measures. The optimal fiber length and the tendon slack length of the scaled model are then optimized (b). The IMU are placed on the model (c) and then used to perform inverse kinematics (d) and obtain joint angles. IMU accelerations are used to detect gait phases (e) and estimate the total 3D GRF (f). The 3D GRF is split into right and left GRFs using the STA (g) and the detected gait phases. The inverse kinematics input is used to track the heel, toes, and CoM of the calcaneus position (*x*, *y*, *z*). Those together with the detected gat phases are used to estimate the CoP (i). Estimated CoP and right and left GRFs are input to the inverse dynamics tool (j) to finally compute the ankle dorsi-plantar flexion torque.

We used the open-source software OpenSim (Delp et al., 2007) and the participant’s height and weight to linearly scale a generic musculoskeletal geometry model (gait 2392) to the person-specific musculoskeletal geometry (Figure 2(a)). A manual scaling factor was defined for each subject as the ratio between the height of the generic musculoskeletal model (168 cm) and the individual height of a participant (Table 1). This yielded a scaled model, from which we derived initial person-specific values of optimal fiber length and tendon slack length for each modeled muscle-tendon unit using a previously developed optimization algorithm (Modenese et al., 2016) (Figure 2(b)). Using the OpenSim IMU placer tool, IMU orientations, represented with quaternions, during a static pose were used to register each IMU sensor to a specific body segment of the scaled and optimized model (Figure 2(c)). Subsequently, the IMU orientations from all the walking tasks were used as input to the Opensim IMU inverse kinematics (IK) tool to obtain knee and ankle joint angles (Al Borno et al., 2022) (Figure 2(d)). We will refer to the joint angles output of the OpenSim IK tool as IMU-based knee and ankle angles. The tool calculated the generalized coordinates of a model that best matched the experimental IMU orientations. Mathematically, this is expressed as a weighted least squares problem, which minimizes the orientation errors (Al Borno et al., 2022).

#### 2.3.2. Gait phases detection

The gait phase detection algorithm was developed to automatically detect initial contact (IC) and terminal contact (TC) events (Figure 2(e)) to subdivide the gait cycle into single and double stance phases. The gait phase detection algorithm is composed of the steps described as follows:

- Foot flat (FF): The foot flat phase, i.e., when the entire leading foot is flat on the ground until the moment before the heel is lifted off the ground, was computed by applying a threshold to the 3D filtered linear velocities, as follows:

(2.1)

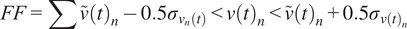

where 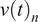 is the linear filtered velocity in the direction 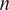 ( 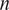 = *x*,*y*,*z*), 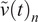 is the mean linear filtered velocity value in each direction, and 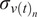 is its standard deviation. Therefore, the FF phase was considered as the set of points in time where the angular velocities in the three directions simultaneously showed a small variation with respect to the mean value that was expected to be close to zero (Rueterbories et al., 2013).

- Signal Vector Magnitude (SVM) of the angular velocities: The SVM of the angular velocities of each foot was calculated as follows (Chang et al., 2016):

(2.2)

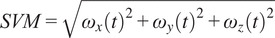

where t is the time step, 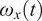 , 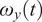 , and 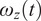 are the filtered angular velocities of in the anterior-posterior, vertical, and mediolateral directions, respectively.

**Figure 3.**
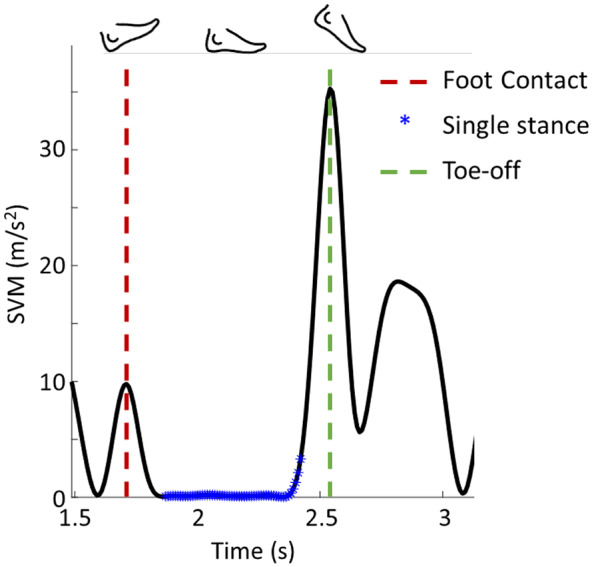
Identification of foot contact, singles stance, and toe-off from signal vector magnitude (SVM).
- Terminal Contact (TC): SVM is relatively large when the foot is at TC in the swing phase (Patterson & Caulfield, 2011) due to the foot moving off the ground during the initial swing. Therefore, we detected as TC point the local maximum right after the end of the FF phase (Figure 3).

- Initial contact (IC): we assumed the SVM was zero, or close to zero when the entire foot is in contact with the ground (FF), that is, when there is no foot rotation and therefore none or almost none angular velocity (Rueterbories et al., 2013). In general, during the IC, a sharp spike in the foot acceleration is easily visible. This then levels out to the FF (Patterson & Caulfield, 2011). Therefore, we defined the IC moment as the local maximum right before the beginning of the FF phase (Figure 3).

These steps were applied to the acceleration of each foot to find the IC and the TC events of both impaired and contralateral sides. Once these four events (IC and TC impaired side, and IC and TC contralateral side) for each gait cycle were detected, the gait phases were defined as follows:

- double stance phase 1: from IC impaired side to TC contralateral side.
- single stance: from TC contralateral side to IC contralateral side.
- double stance phase 2: from IC contralateral side to TC impaired side.
- swing phase: from TC impaired side to following IC impaired side.

#### 2.3.3. Estimation GRFs

The sensors’ local linear accelerations were first expressed in the origin frame (2.3) and then rotated to be aligned to the origin OpenSim frame (2.4). To compute the total external force, we used the inverted pendulum model of gait, which assumes that (1) the GRF accelerates the center of mass (CoM) and opposes gravity, and (2) the CoM is bound to the pelvis. By knowing the kinematics and inertial properties of the segments of the musculoskeletal model and assuming that the feet are the only contact with the ground, we estimated the total 3D GRF using Newton’s equations of motion 2.5 (Figure 2(f)).(2.3)

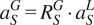


(2.4)

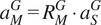


(2.5)

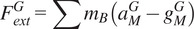


(2.6)

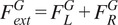

 where 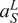 is the sensor (S) local (L) linear acceleration, 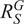 is the rotation matrix to the global sensor’s frame obtained from the sensor orientations, 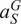 is the sensor’s global linear acceleration, 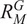 is the rotation matrix to the model frame representing a − 90-deg rotation around the x-axis, 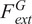 is the 3D total external force in the global model frame, 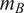 mass of each body segment (the pelvis contains the mass of the entire body except for the impaired leg and the contralateral foot), 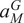 is the linear acceleration of the single segment, 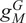 is the gravity, and 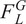 and 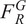 are the left and right 3D GRFs, all in the global model frame.

To split the total 3D GRF into the right and left sides 2.6, we use the smooth transition assumption (STA) (Karatsidis et al., 2017) (Figure 2(g)). It assumes that during the double stance, there is a smooth load transition from the trailing foot to the leading foot. The behavior of the components of the STA functions, in all dimensions, used in this work is described in detail in (Ren et al., 2008). We referred to the obtained GRFs as IMU-based GRFs.

#### 2.3.4. Estimation of Center of Pressure (CoP)

**Figure 4.**
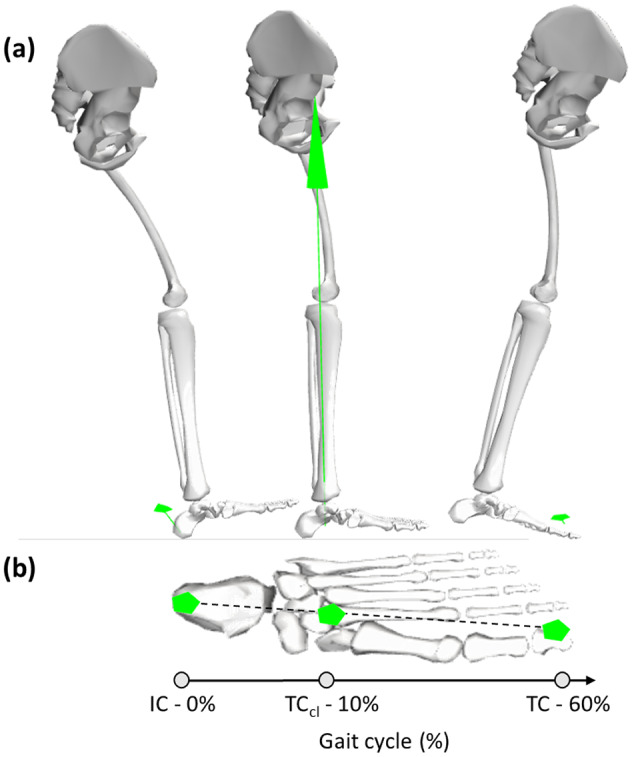
Center of pressure (CoP) displacement from foot contact (FC) to Toes-off (TO). The CoP and the GRFs (green arrows) are shown in three instants of the gait cycle (FC, contralateral toes-off - TO_cl_, and TO) in two different views: (a) lateral view, (b) from the top.

To estimate the center of application of the external force (Figure 2(i)), i.e., CoP, during gait, we assumed that its position traveled linearly from the heel (at IC) to the toes (at TC), and it was always within the base of support as reported by (De Cock et al., 2008). We defined three points in the foot in the generic unscaled model: the heel, the calcaneus CoM, and the end of the toe segment. The calcaneus CoM position was already defined in the musculoskeletal model, while the position of the heel and the end of the toe were manually defined. Their positions (*x*, *y*, *z*) with respect to the calcaneus CoM in the generic musculoskeletal model are the following: heel = [0.00363314, 0.00830251, −0.00715492] m, toe = [0.231826, −0.0107138, −0.0071549] m. However, such positions were scaled to each participant’s foot dimensions using the manual scaling factor computed during the scaling of the generic musculoskeletal geometry (Section 2.3.1). We capture the position of these points during gait by using the Point Kinematics OpenSim tool (Schutte et al., 1993) (Figure 2(h)). For each gait cycle, the CoP in the vertical direction was set to zero. For the anterior-posterior (*x*) and the mediolateral directions (*z*), the CoP position starts at the IC moment from the position of the pre-defined heel point, and it travels during the double stance 1 and finally reaches CoM of the calcaneus (Figure 4) when in the contralateral TC. Finally, during the FF and double stance 2 phases, the CoP translated from the calcaneus CoM to the position of the pre-defined toe point.

### 2.4. Laboratory system

The open-source software OpenSim (Delp et al., 2007) and experimental marker trajectories from a static task were used to scale a generic musculoskeletal geometry model (gait 2392). The generic model is equipped with virtual markers positioned at corresponding anatomical locations to the experimental markers. Scaling adjustments were made to the dimensions of each segment in the generic model to match the distances between the virtual markers to those of the experimental. Subsequently, from the scaled model, the optimal fiber length and tendon slack length of each muscle-tendon unit were optimized with the same Matlab tool as described in Section 2.3.1. Optimized scaled models and experimental marker trajectories during the walking tasks were used to solve IK and obtain reference joint angles. The Opensim IK tool iterates through each time frame of experimental marker data, adjusting the optimized scaled model’s pose to align closely with the experimental marker for that specific time step. This alignment is achieved by minimizing the sum of weighted squared errors of markers, ensuring the model closely replicates the observed movements captured by the markers (Delp et al., 2007).

GRF and CoP were directly measured from the double force plates, and we refer to them as reference GRFs and reference CoP.

### 2.5. Inverse dynamics (ID)

IMU-based joint angles, GRFs, and CoP trajectories, as well as reference joint angles, GRFs, and CoP trajectories, were input to the inverse dynamics Opensim tool (Figure 2(j)) to obtain IMU-based and reference ankle dorsi-plantar flexion torques, respectively.

### 2.6. EMG-driven musculoskeletal modeling

We employed an EMG-driven musculoskeletal model (Durandau et al., 2018) to estimate ankle dorsi-plantar flexion torques.

**Figure 5.**
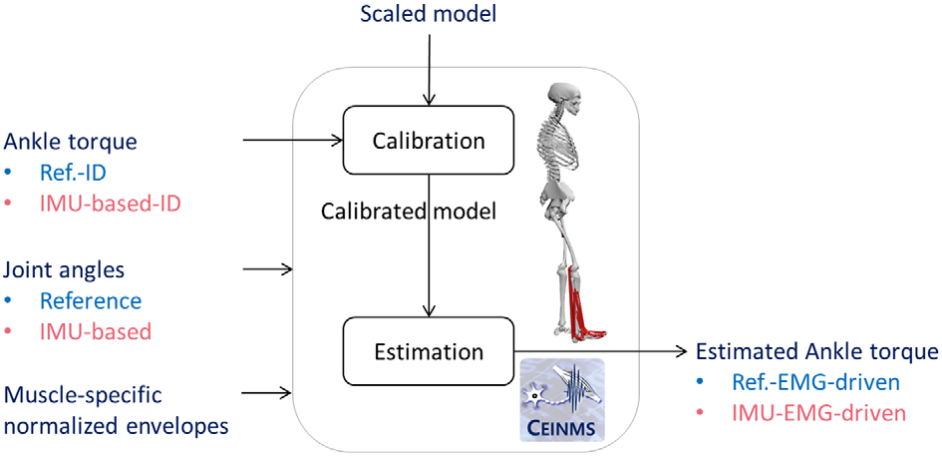
EMG-driven modeling pipeline comprising a calibration process to optimize the muscle-tendon unit parameters and an estimation process to estimate ankle torque from muscle-specific normalized envelopes and joint angles.

For each participant, we carried out a calibration process (Figure 5) to optimize the musculoskeletal model parameters (optimal fiber length, tendon slack length, strength coefficient, and shape factor of each muscle-tendon unit) that do not vary linearly across individuals to match each individual’s force-generating capacity. This optimization aimed to minimize the mean squared error between the reference and EMG-driven model estimated torques normalized by the variance of the reference torque over the first gait cycle performed at a comfortable walking speed. We performed two calibrations for each participant using just one gait cycle of a comfortable walking speed. The calibration input torque was either the reference or IMU-based ankle dorsi-plantar flexion torque (Figure 5). After calibration, the person-specific EMG-driven musculoskeletal model was used to estimate muscle-tendon force and resulting ankle dorsi-plantar flexion torque using input joint angles (reference and IMU-based) and normalized EMG linear envelopes (Figure 5) during novel trials and walking tasks that were not used for the model calibration 5. We referred to the EMG-driven estimated torque driven by reference and IMU-based data as reference-EMG-driven and IMU-EMG-driven torques, respectively.

### 2.7. Validation procedures

We performed two tests to evaluate kinematic and kinetic data retrieved by laboratory and wearable systems and from ankle dorsi-plantar flexion torques estimated using laboratory- or wearable-derived signals and an EMG-driven model.

Test 1 evaluated the first two hypotheses, i.e., removing the need for laboratory-based technologies and computing knee and ankle angles and ID ankle dorsi-plantar flexion. We compared the output of the laboratory (camera-based motion tracking system and force plates) and wearable (five IMUs) systems at the level of knee and ankle flexion-extension angles, 3D GRFs, 3D CoP trajectory, and ankle dorsi-plantar flexion torques derived from inverse dynamics. For each post-stroke individual and all walking speeds, the coefficient of determination (*R*^2^) for shape similarity and the root mean square error (RMSE) for amplitude similarity were computed between the laboratory system (reference) and the wearable system (IMU-based) outputs.

Test 2 evaluated our third hypothesis, *which is* the capability of the proposed wearable technology (EMG-sensorized garment and five IMUs) to estimate ankle dorsi-plantar flexion torques via a musculoskeletal model driven by IMU-based and EMG signals. We calibrated the person-specific musculoskeletal model in two different ways, i.e., Ref-calibrated and IMU-calibrated (Section 2.6), and we obtained two ankle dorsi-plantar flexion torque estimates: (1) ankle torque estimated using a Ref-calibrated musculoskeletal model driven by lab-based joint angles and EMG signals (Ref-EMG-driven), and (2) ankle torque estimated using an IMU-calibrated musculoskeletal model driven by IMU-based joint angles and EMG signals (IMU-EMG-driven). We compared the reference ankle dorsi-plantar flexion torque (Ref-ID) with respect to the ankle dorsi-plantar flexion torque output of the two musculoskeletal models, i.e., Ref-EMG-driven and IMU-EMG-driven. For each post-stroke individual and all walking speeds, *R*^2^ for shape similarity and RMSE for amplitude similarity were computed.

Both tests were performed on a control group of nine healthy participants and a group of four post-stroke individuals. The information about the healthy participants, experimental procedure, and test results is presented in Supplementary Material.

## 3. Results

In the following section, only the results of post-stroke participants are shown. In Supplementary Material are presented the test results on healthy participants.

### 3.1. Test 1

*R*^2^ and RMSE values were computed to evaluate the shape and amplitude similarity between reference and estimated knee and ankle angles, 3D GRFs, 3D CoP trajectory, and ID ankle dorsi-plantar flexion torque.

**Figure 6.**
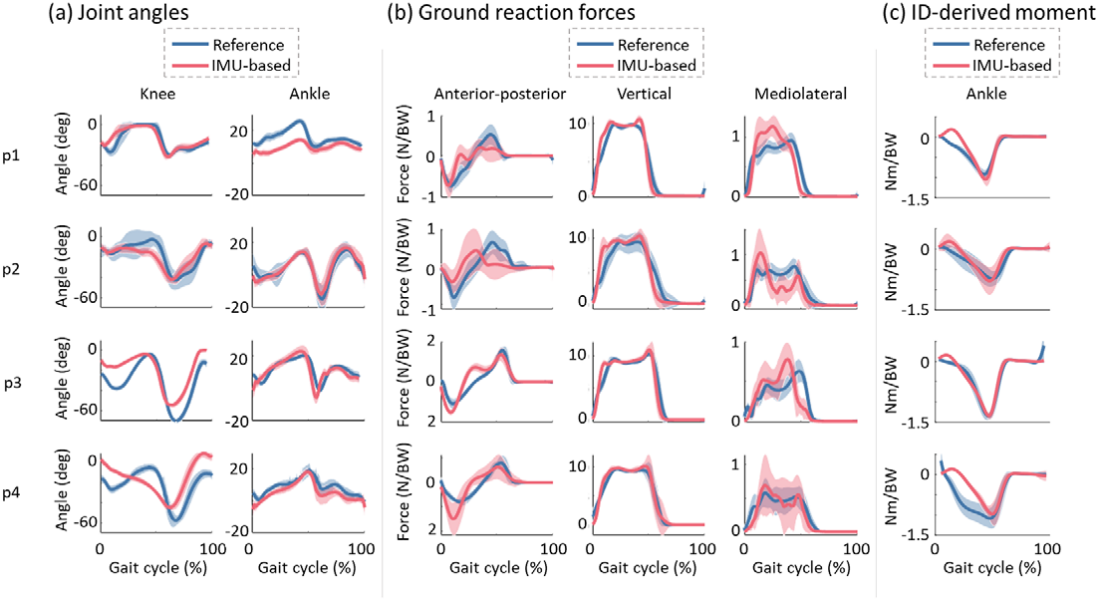
Comparison between reference (in blue) and estimated (in red) joint angles (a), 3D GRFs (b) and inverse dynamics-derived ankle dorsi-plantar flexion torques (c) averaged across all gait cycles for each post-stroke individual walking at a self-selected comfortable speed. The solid line represents the mean values, while the shaded area is the standard deviation.

**Figure 7.**
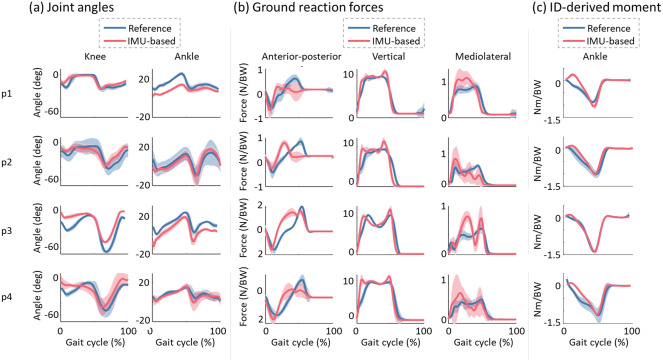
Comparison between reference (in blue) and estimated (in red) joint angles (a), 3D GRFs (b), and inverse dynamics-derived ankle dorsi-plantar flexion torques (c) averaged across all gait cycles for each post-stroke individual walking at a self-selected fast speed. The solid lines represent the mean values, while the shaded area is the standard deviation.

Figures 6 and 7 show the comparison between reference and estimated leg joint angles (Figures 6(a) and 7(a)), 3D GRFs (Figures 6(b) and 7(b)), and ID ankle dorsi-plantar flexion torques (Figures 6(c) and 7(c)) averaged across all gait cycles for self-selected comfortable and fast walking speed for each post-stroke individual, respectively. These figures show a close match in amplitude and shape between reference and estimated joint angles and vertical GRFs. However, a small offset is visible in the estimated knee angle for participant 3 and the estimated ankle angle for participants 1 and 3. For anterior-posterior and mediolateral GRFs, the comparison showed more visible average trend differences. For the ID ankle dorsi-plantar flexion torques, all participants show a close match with the negative plantar-flexion peak and close shape similarity in the second half of the gait cycle. However, during the first half of the gait cycle, a more marked difference in the shapes is visible. Reference torque shows negative values from the IC (0%) to the TC (negative peak), while the estimated torque shows a small dorsi flexion, *i.e.*, positive values, during the first double support phase.

**Figure 8.**
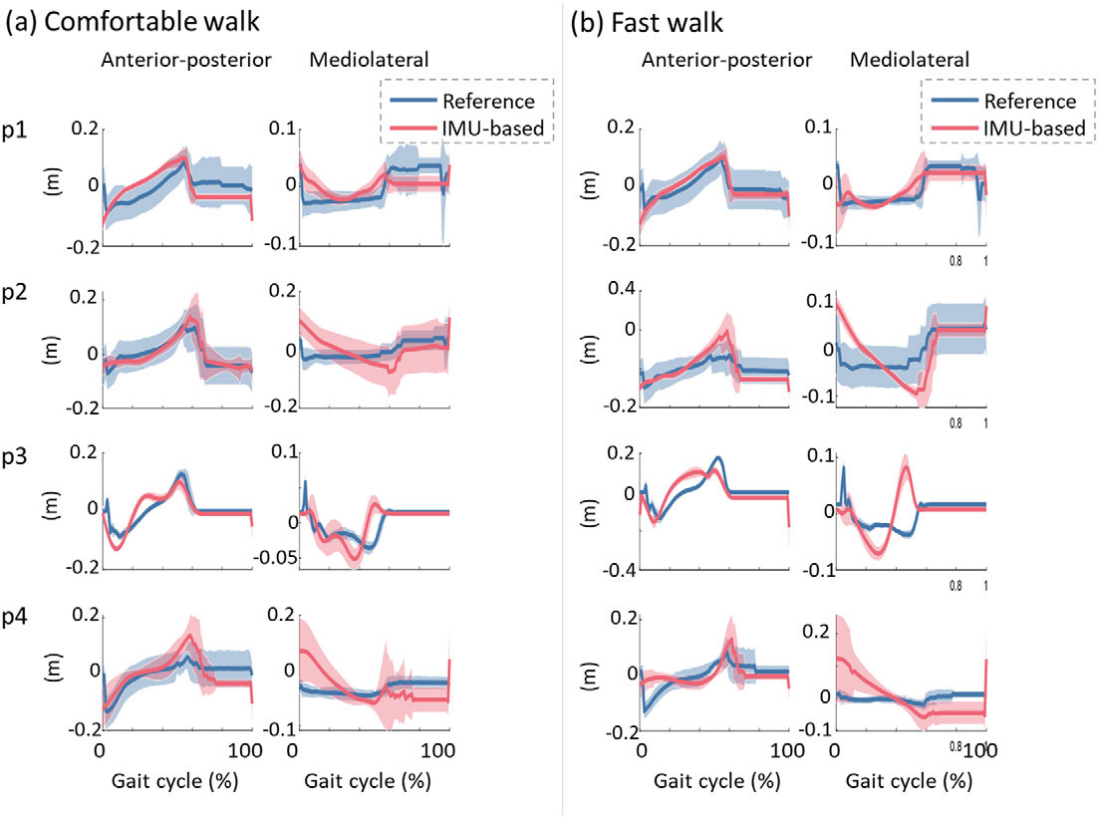
Comparison between reference (in blue) and estimated (in red) center of pressure (CoP) averaged across all gait cycles for each post-stroke individual walking at self-selected comfortable (a) and fast (b) speeds. The solid lines represent the mean values, while the shaded area is the standard deviation.

Figure 8 shows the comparison between reference and estimated CoP trajectory in the two directions, i.e., anterior-posterior and mediolateral, during walking at self-selected comfortable (Figure 8(a)) and self-selected fast speeds (Figure 8(b)). For all speeds, the estimated CoP trajectory in the anterior-posterior direction showed a similar trend, i.e., a positive slope, with the reference CoP trajectory. A more marked difference is visible for the CoP trajectory in the mediolateral direction, especially for participants 2 and 4 during both walking speeds.

Tables 2 and 3 present the *R*^2^ and RMSE values between reference and estimated kinematics (knee and ankle angles) and kinetic (3D GRFs and ID ankle dorsi-plantar flexion torque) data for each post-stroke individual. For the knee angles, *R*^2^ and RMSE values averaged across all participants, and all walking speeds ranged between 0.47 and 0.86 with a mean (± std) of 0.70 ± 0.13, and between 5 and 19 deg with a mean (± std) of 12 ± 4 deg, respectively. For the ankle angles, *R*^2^ and RMSE values averaged across all participants, and all walking speeds ranged between 0.33 and 0.76 with a mean (± std) of 0.53 ± 0.15, and between 5 and 11 deg with a mean (± std) of 8 ± 2 deg, respectively.

**Table 2.** *R*^2^ values between experimental and estimated (IMU based) flexion-extension angle of knee and angle, 3D GRFs, 3D CoP and ID-derived ankle dorsi-plantar flexion torques during walking at a self-selected comfortable and self-selected fast speed for post-stroke individuals

Comf W.	Joint angles		GRFs			CoP		ID-torque Ankle
	Knee	Ankle	AP	V	ML	AP	ML	
p1	0.68 ± 0.19	0.50 ± 0.20	0.45 ± 0.25	0.94 ± 0.04	0.77 ± 0.10	0.31 ± 0.33	0.25 ± 0.20	0.87 ± 0.06
p2	0.47 ± 0.24	0.33 ± 0.16	0.20 ± 0.23	0.72 ± 0.29	0.21 ± 0.15	0.42 ± 0.35	0.15 ± 0.16	0.76 ± 0.06
p3	0.81 ± 0.11	0.76 ± 0.14	0.67 ± 0.10	0.92 ± 0.06	0.27 ± 0.09	0.67 ± 0.06	0.26 ± 0.11	0.68 ± 0.21
p4	0.68 ± 0.31	0.46 ± 0.29	0.54 ± 0.18	0.87 ± 0.15	0.25 ± 0.15	0.33 ± 0.32	0.20 ± 0.24	0.51 ± 0.23
**Mean**	**0.66** ± **0.12**	**0.51** ± **0.16**	**0.46** ± **0.17**	**0.86** ± **0.09**	**0.38** ± **0.23**	**0.43** ± **0.15**	**0.21** ± **0.05**	**0.71** ± **0.13**

Fast W.	Joint angles		GRFs			CoP		ID-torque Ankle
	Knee	Ankle	AP	V	ML	AP	ML	
p1	0.77 ± 0.09	0.47 ± 0.15	0.36 ± 0.23	0.93 ± 0.08	0.81 ± 0.08	0.38 ± 0.27	0.35 ± 0.22	0.89 ± 0.05
p2	0.54 ± 0.21	0.72 ± 0.24	0.19 ± 0.15	0.84 ± 0.08	0.15 ± 0.16	0.37 ± 0.30	0.29 ± 0.22	0.63 0.23
p3	0.77 0.06	0.65 ± 0.09	0.25 ± 0.13	0.37 ± 0.21	0.13 ± 0.12	0.44 ± 0.11	0.05 ± 0.04	0.73 ± 0.07
p4	0.86 ± 0.12	0.37 ± 0.18	0.50 ± 0.12	0.46 ± 0.46	0.08 ± 0.05	0.20 ± 0.09	0.21 ± 0.19	0.57 ± 0.26
**Mean**	**0.7** ± **0.12**	**0.55** ± **0.14**	**0.33** ± **0.12**	**0.65** ± **0.24**	**0.29** ± **0.30**	**0.35** ± **0.09**	**0.22** ± **0.11**	**0.70** ± **0.12**

*Abbreviations:* AP = anterior–posterior; V = vertical; ML = mediolateral.

**Table 3.** RMSE values between experimental and estimated (IMU based) flexion-extension angle of knee and angle, 3D GRFs, 3D CoP and ID-derived ankle dorsi-plantar flexion torques during walking at a self-selected comfortable and sel-selected fast speed for post-stroke individuals

Comf W.	Joint angles		GRFs			CoP		ID-torque Ankle (Nm/kg)
	Knee (deg)	Ankle (deg)	AP (N/kg)	V (N/kg)	ML (N/kg)	AP (m)	ML (m)	
p1	6.27 ± 1.81	7.40 ± 1.43	0.45 ± 0.16	1.14 ± 0.31	0.79 ± 0.45	0.07 ± 0.03	0.04 ± 0.01	0.12 ± 0.06
p2	18.72 ± 28.16	11.47 ± 7.58	0.58 ± 0.09	1.70 ± 0.40	0.63 ± 0.18	0.07 ± 0.03	0.07 ± 0.06	0.27 ± 0.08
p3	14.09 ± 1.20	7.58 ± 1.55	0.84 ± 0.48	1.21 ± 0.40	0.47 ± 0.19	0.03 ± 0.01	0.02 ± 0.01	0.39 ± 0.24
p4	12.36 ± 6.41	8.40 ± 4.74	0.69 ± 0.21	1.53 ± 0.92	0.48 ± 0.20	0.07 ± 0.04	0.07 ± 0.03	0.47 ± 0.12
**Mean**	**12.86** ± **4.46**	**8.71** ± **1.63**	**0.64** ± **0.14**	**1.39** ± **0.23**	**0.59** ± **0.13**	**0.06** ± **0.01**	**0.05** ± **0.02**	**0.29** ± **0.15**

Fast W.	Joint angles		GRFs			CoP		ID-torque Ankle (Nm/kg)
	Knee (deg)	Ankle (deg)	AP (N/kg)	V (N/kg)	ML (N/kg)	AP (m)	ML (m)	
p1	5.51 ± 1.24	7.05 ± 0.62	0.32 ± 0.10	1.17 ± 0.48	0.24 ± 0.05	0.06 ± 0.02	0.03 ± 0.01	0.11 ± 0.02
p2	11.16 ± 3.49	5.07 ± 2.54	0.52 ± 0.13	2.27 ± 1.31	0.50 ± 0.12	0.13 ± 0.02	0.09 ± 0.04	0.45 ± 0.51
p3	15.37 ± 1.04	5.04 ± 0.86	1.53 ± 1.11	4.46 ± 1.20	0.46 ± 0.13	0.06 ± 0.01	0.04 ± 0.01	0.27 ± 0.13
p4	10.73 ± 1.12	8.70 ± 1.15	0.38 ± 0.08	3.63 ± 2.41	0.42 ± 0.10	0.06 ± 0.02	0.08 ± 0.01	0.40 ± 0.13
**Mean**	**10.69** ± **3.50**	**6.46** ± **1.53**	**0.69** ± **0.49**	**2.88** ± **1.26**	**0.40** ± **0.10**	**0.08** ± **0.03**	**0.06** ± **0.03**	**0.31** ± **0.13**

*Abbreviations:* AP = anterior–posterior; V = vertical; ML = mediolateral

For the 3D GRFs, *R*^2^ and RMSE values across all post-stroke individuals and walking speeds were 0.39 ± 0.16 and 0.66 ± 0.36 N/kg in the anterior-posterior direction, 0.76 ± 0.29 and 2.01 ± 1.60 N/kg in the vertical direction, and 0.33 ± 0.27 and 0.50 ± 0.27 N/kg in the mediolateral direction, respectively. For the CoP, *R*^2^ and RMSE values averaged across all post-stroke individuals and for all walking speeds were 0.39 ± 0.13 and 11.75 ± 21.2 mm in the anterior-posterior direction, and 0.22 ± 0.09 and 8.11 ± 11.65 m in the mediolateral direction, respectively. For the ID ankle dorsi-plantar flexion torques, *R*^2^ and RMSE values averaged across all post-stroke individuals and for all walking speeds were 0.71 ± 0.13 and 0.30 ± 0.14 Nm/kg, respectively.

### 3.2. Test 2

**Figure 9.**
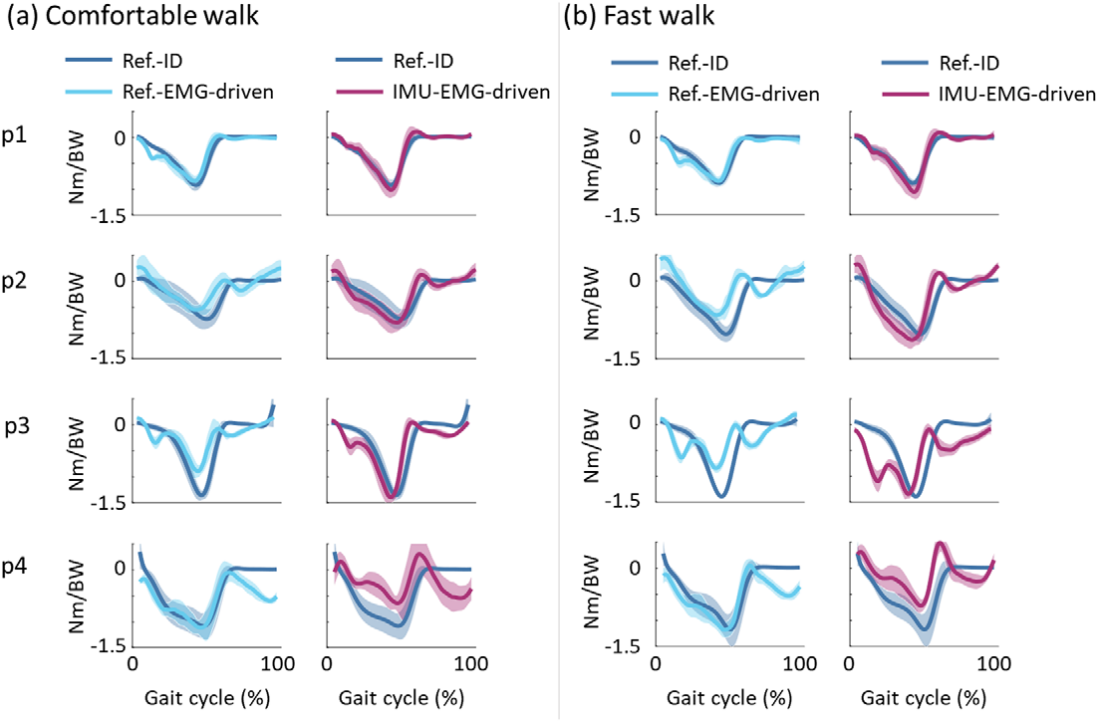
Comparison between reference and EMG-driven estimated ankle dorsi-plantar flexion torques. For each post-stroke individual, across all gait cycles of self-selected comfortable (a) and fast (b) walking speed, reference ankle torque (Ref-ID, dark blue line) is compared with (a) laboratory-derived signals- and EMG-driven torque (Ref-EMG-driven, light blue line), and IMU-based signal- and EMG-driven torque (IMU-EMG-driven, purple line).

Figure 9 shows the comparison between reference ID torque (ID ankle dorsi-plantar flexion torque informed by lab-bound signals, Ref-ID) and output ankle dorsi-plantar flexion torque output of two EMG-driven models: (1) lab-bound signals-EMG-driven model and (2) a fully wearable (IMU and EMG)-driven model. The torques were averaged across all gait cycles for each post-stroke individual walking at self-selected comfortable (Figure 9(a)) and fast (Figure 9(b)) speeds. Participant 1 presents estimated torques (Ref-EMG-driven and IMU-EMG-driven) with a similar trend with respect to the reference ID torque. Participants 2 and 3 present IMU-EMG-driven torque with a visible higher shape similarity to the reference ID torque with respect to the Ref-EMG-driven torque. On the contrary, participant 4 presented better EMG-driven ankle dorsi-plantar flexion torque when the musculoskeletal model was calibrated and driven by lab-derived signals (Ref-EMG-driven).

Table 4 shows the *R*^2^ and RMSE values for reference ID torque and the ankle dorsi-plantar flexion torque output of two EMG-driven models, i.e., Ref-EMG-driven and IMU-EMG-driven, for each post-stroke individual and for all walking speeds. *R*^2^ and RMSE values averaged across all post-stroke individuals, and speeds were 0.60 ± 0.19 and 0.39 ± 0.19 Nm/kg between reference ID and Ref-EMG-driven ankle dorsi-plantar flexion torques, 0.65 ± 0.21 and 0.35 ± 0.16 Nm/kg between reference ID and IMU-EMG-driven, respectively.

**Table 4.** *R*^2^ and normalized (by body weight) RMSE values between reference (Ref-ID) and both Ref-EMG-driven and IMU-EMG-driven ankle dorsi-plantar flexion torques across all post-stroke individuals walking at self-selected comfortable and self-selected fast speeds

*R*^2^	Comfortable walk		Fast walk	
	Ref-EMG-driven	IMU-EMG-driven	Ref-EMG-driven	IMU-EMG-driven
p1	0.81 ± 0.10	0.92 ± 0.03	0.76 ± 0.08	0.91 ± 0.03
p2	0.65 ± 0.24	0.77 ± 0.08	0.74 ± 0.27	0.62 ± 0.28
p3	0.68 ± 0.27	0.67 ± 0.27	0.22 ± 0.16	0.64 ± 0.14
p4	0.50 ± 0.17	0.32 ± 0.14	0.45 ± 0.30	0.34 ± 0.17
**Mean**	**0.66** ± **0.11**	**0.67** ± **0.22**	**0.54** ± **0.23**	**0.63** ± **0.20**

RMSE (Nm/kg)	Comfortable walk		Fast walk	
	Ref-EMG-driven	IMU-EMG-driven	Ref-EMG-driven	IMU-EMG-driven
p1	0.18 ± 0.03	0.13 ± 0.03	0.20 ± 0.02	0.13 ± 0.02
p2	0.23 ± 0.08	0.19 ± 0.05	0.39 ± 0.51	0.49 ± 0.55
p3	0.37 ± 0.24	0.40 ± 0.27	0.82 ± 0.05	0.39 ± 0.11
p4	0.46 ± 0.16	0.54 ± 0.12	0.45 ± 0.13	0.53 ± 0.10
**Mean**	**0.31** ± **0.11**	**0.32** ± **0.17**	**0.47** ± **0.23**	**0.39** ± **0.15**

## 4. Discussion

We presented a wearable technology that employed a multi-channel EMG-sensorized garment and an automated muscle localization technique for unsupervised computation of muscle-specific activations along with five IMUs for the computation of joint kinetics and kinematics during walking at different speeds. This was done for the first time in the present work on both healthy (Supplementary Material) and post-stroke participants. The proposed wearable technology removes the need for laboratory-based technologies, such as force plates and camera-based tracking systems, and lengthy procedures for manual muscle localization and manual sensor placement. Furthermore, the wearable sensors and automated processing techniques were combined with an EMG-driven musculoskeletal model for the non-invasive estimation of ankle dorsi-plantar flexion torque as the result of the forces exerted by the underlying muscles. This can allow the estimation of muscle kinetics and resulting joint torque for motor impairment monitoring in time- and space-constrained environments such as rehabilitation sessions in clinics. Furthermore, wearable technologies can facilitate the study of human movement biomechanics outside of the controlled lab environment.

The results showed the capability of the proposed wearable technology to extract reasonable joint kinematics and kinetics (Test 1) in both healthy (Supplementary Material) and post-stroke participants (Section 3.1). Despite having lower accuracy than the IMU-based ID-derived ankle dorsi-plantar flexion torque, when IMU-based estimation of kinetics and kinematics were combined with EMG signals to drive a musculoskeletal model, reasonable estimates of ankle dorsi-plantar flexion torques (Test 2) during different walking speeds for healthy (Supplementary Material) and post-stroke individuals (Section 3.2) were obtained.

Our first test showed that IMU-based IK could extract accurate knee angles in healthy (*R*^2^ = 0.92 ± 0.08, RMSE = 6 ± 3 deg, Supplementary Figure 1(a)) and reasonable estimates in post-stroke individuals (*R*^2^ = 0.70 ± 0.13, RMSE = 6 ± 19 deg, Figures 6(a) and 7(a)) during different walking speeds. These results were in line with previously reported results (Weygers et al., 2020). However, for ankle angles, the accuracy decreases in both populations and particularly for post-stroke individuals (*R*^2^ = 0.53 ± 0.15, RMSE = 8 ± 2 deg). This might be due to the lower range of motion of post-stroke individuals as well as the slower walking speed with respect to healthy participants (Carmo et al., 2012), hence making the movement less pronounced and more difficult for inertial sensors to capture accurately (Revi et al., 2020). Furthermore, the calibration process that registered each IMU to a specific body segment was based on the assumption that the participant was in a specific neutral pose during the static standing trial (Section 2.3.1), i.e., in a neutral pose resembling the default pose of the generic OpenSim model. This becomes more challenging in post-stroke individuals that present greater asymmetries between the paretic and non-paretic sides (Titianova & Tarkka, 1995) and hence are not able to stand in a neutral position with weight distributed equally between both sides.

The results on GRFs estimated from IMUs’ linear accelerations showed that the vertical forces are the most accurate in both healthy (mean *R*^2^ > 0.91, Supplementary Figure 1(b), Supplementary Table 1) and post-stroke individuals (mean *R*^2^ > 0.65, Figures 6(b), 7(b), Tables 2, and 3). As observed in the literature, the estimation of the external forces in the anterior-posterior and mediolateral directions is more challenging (Ren et al., 2008; Karatsidis et al., 2017), even more in post-stroke individuals (Tables 2 and 3) where movements are slower and less marked (Revi et al., 2020).

The less accurate results were observed for the IMU-based 3D CoP trajectory. With the use of the simple assumption of the CoP traveling from the heel, as the IC point, to the toes at the TC moment, the reported estimation showed a similar trend in the anterior-posterior CoP trajectory in both populations (Supplementary Figures 2 and 8). In the mediolateral direction, the CoP trajectory showed a better estimation for the post-stroke individuals (*R*^2^ = 0.22 ± 0.09) with respect to the healthy participants (*R*^2^ = 0.11 ± 0.17). This might be due to the slower walking speed and range of motion caused by motor impairment (Carmo et al., 2012).

Nevertheless, the IMU-based joint angles, 3D GRFs, and especially CoP trajectory, translated in IMU-based ID ankle dorsi-plantar flexion torques with a promising degree of accuracy with respect to reference ID ankle dorsi-plantar flexion torque, in both healthy (Supplementary Table 1) and post-stroke individuals (Tables 2 and 3). While the negative plantar flexion peak is well detected in magnitude and timing, during the single stance phase the shape of the IMU-based ID ankle dorsi-plantar flexion torques presented some discrepancies with respect to the reference ID ankle dorsi-plantar flexion torque (Supplementary Figure 3). This might be due to the less accurate estimation of the IMU-based CoP trajectory built on a simple assumption (Section 2.3.4, Supplementary Tables 1, 2, and Table 3). The assumption used to compute the CoP in the anterior-posterior and mediolateral directions cannot accurately track changes of the CoP, especially in abnormal gait where the IC might not always be at the heel and the CoP might not travel from the back to the front of the foot (Jamshidi et al., 2010).

IMU-based knee and ankle angles and IMU-based ID ankle dorsi-plantar flexion torques are finally used to inform an EMG-driven musculoskeletal model in order to estimate ankle dorsi-plantar flexion torque resulting from the underlying muscle forces. The musculoskeletal model parameters (Section 2.6) were calibrated two different times using a single gait cycle of the comfortable walking speed using two different input joint kinetics and kinematics, i.e., reference and IMU-based. We were then able to calibrate the musculoskeletal model parameters for the specific participant and estimate ankle dorsi-plantar flexion torque during unseen trials and unseen walking speeds, i.e., not used during the model calibration. With test 2, we show that with a standard setup, i.e., in a laboratory with cameras and force plates, reference-EMG-driven ankle dorsi-plantar flexion torques were computed with a promising accuracy (Table 4) with respect to the reference ID ankle dorsi-plantar flexion torque (Ref-ID). When using a fully wearable setup (IMUs and EMG-sensorized garment) the estimation of ankle dorsi-plantar flexion torques via the IMU-EMG-driven model showed higher estimation errors compared to ankle dorsi-plantar flexion torque retrieved via IMU-based inverse dynamics. However, the IMU-EMG-driven musculoskeletal model outputs ankle dorsi-plantar flexion torque with marginally lower estimation errors than the one obtained via the reference-EMG-driven model (Table 4). The shape of the IMU-EMG-driven ankle dorsi-plantar flexion torque during the single stance phase (Figure 9) better resembles the profile of the reference ID ankle dorsi-plantar flexion torque compared to the IMU-based ID ankle dorsi-plantar flexion torque (Figures 6 and 7). This might suggest that the EMG-driven model can compensate for the lower accurate IMU-based joint angles and ID-derived ankle dorsi-plantar flexion torques during the calibration process. During the calibration, muscle-tendon unit (MTU) parameters were tuned to best match the input ankle dorsi-plantar flexion torque (input Ref-ID for Ref-EMG-driven model and input IMU-based ID for IMU-EMG-driven model). The EMG-driven output ankle dorsi-plantar flexion torques are dictated by both EMG signals and joint angles. IMU-EMG-driven ankle dorsi-plantar flexion torques were less sensitive to changes in MTU parameters than to the driving neural signal, i.e., muscle-specific EMGs. Therefore, after pre-calibrating MTU parameters (Section 2.3.1), the narrow ranges for MTU parameters (around 5% or the values found during scaling - Section 2.3) did not substantially affect the calibrated model, and therefore we were still able to obtain IMU-EMG-driven ankle dorsi-plantar flexion torque comparable to the reference ID ankle dorsi-plantar flexion torque.

While IMUs and a person-specific multi-dynamic model obtained the most accurate body kinematics and kinetics, this approach restricts musculoskeletal assessment to the joint level. For individuals with neurological impairment such as post-stroke, relying solely on kinetics and kinematics is insufficient for a comprehensive clinical assessment since the cause of the altered motion, i.e., the muscles, is not directly assessed. To address this limitation, introducing EMGs, particularly via an easy-to-wear EMG-embedded sleeve combined with an automated muscle localization algorithm and EMG-driven musculoskeletal modeling, becomes crucial. This setup helps identify abnormal muscle activity, such as co-contraction or spasticity, and muscle forces and enhances the assessment of neurologically impaired individuals while estimating ankle dorsi-plantar flexion torque via IMU-informed inverse dynamics.

This study includes limitations that should be addressed in future works. The estimation of CoP was based on a simple assumption (Section 2.3.4). Future work should improve the estimation of CoP via the use of portable force sensors or advanced machine-learning techniques in order to improve the computation of joint moments via ID. This solution could also help translate the proposed technology to individuals not able to stand or walk independently. Since one of the assumptions for the GRF estimation during walking stated that the participant needed to walk without external aid, with the feet as the only contact with the ground, the IMU-based estimation of GRFs cannot give good estimates in the case of individuals walking with external support, e.g., crutches. The IMU calibration process assumed that the IMUs were attached to the CoM of each body segment. However, this is not true in reality since the sensors are worn and placed on the skin. Future work should improve IMU calibration to lead to higher accuracy in the estimation of joint kinematics and kinetics. Future work should investigate the reliability of gait phase detection during abnormal gait. An accurate gait phase detection is crucial for the accurate split of estimated GRFs on the left and right sides. In the current work, we focused on the estimation of ankle dorsi-plantar flexion torque just in the sagittal plane. More degrees of freedom, as well as more joints, should be included in future works. Furthermore, GRFs and ankle dorsi-plantar flexion torque were computed during walking tasks. More movements should be added to evaluate the generalizability of the EMG-driven model to estimate ankle moments in various dynamics and isometric tasks. To make the proposed wearable technology fully portable and ready for everyday clinical use, cables should not be used. Our solution still includes a long cable (10 m) for the synchronized recording of IMU and EMG signals. This limits the use of the proposed wearable technology for completely unconstrained movement and application. By replacing optical fiber with a Wi-Fi connection for the EMG amplifier, it would allow a cable-free technology. However, further study should assess the impact of EMG data loss due to Wi-Fi communication. The validation metrics used to compare the reference and the IMU-based output were based on *R*^2^ and RMSE for shape and amplitude similarity, respectively. However, this study included nine healthy participants and four male post-stroke individuals. To augment the statistical power, future work should include a larger cohort of healthy participants and post-stroke individuals, ensuring high inter-subject variability. Considering the significant age and weight differences between the healthy control group and post-stroke individuals, future work should include more diverse participants in both groups. Additionally, a larger variety of FAC scores across post-stroke participants should be investigated to enhance the generalizability of the methodology to a wider range of motor impairments. Finally, future research should assess the clinical relevance and practical application of our proposed methodology to diverse injured populations with a variety in levels of severity, e.g., SCI individuals, elderly, or injured athletes. This would not only enhance the statistical power but also provide a more comprehensive understanding of the methodology’s generalization capacity.

## Conclusion

In the present study, we propose a wearable technology that enables the application of biomechanical gait analysis and EMG-driven musculoskeletal simulations by involving automated and advanced processing techniques with a multi-channel EMG-sensorized garment and five IMU sensors. These open up new avenues for the development of portable gait analysis tools in clinical applications for monitoring musculoskeletal systems and designing personalized interventions.

## Supporting information

Simonetti et al. supplementary material Simonetti et al. supplementary material

## Data Availability

An open-access dataset (https://zenodo.org/record/8360018) was generated from the experiment, providing documentation, data processing code, and processed data of lab and wearable systems sensors as well as extracted muscle activations.

## Supplementary material

The supplementary material for this article can be found at http://doi.org/10.1017/wtc.2024.14.

## References

[r1] Al Borno M, O’Day J, Ibarra V, Dunne J, Seth A, Habib A, Ong C, Hicks J, Uhlrich S and Delp S (2022). OpenSense: An open-source toolbox for inertial-measurement-unit-based measurement of lower extremity kinematics over long durations. Journal of Neuroengineering and Rehabilitation 19, 1–11. URL: https://jneuroengrehab.biomedcentral.com/articles/10.1186/s12984-022-01001-x 35184727 10.1186/s12984-022-01001-x PMC8859896

[r2] Barbeau H, Ladouceur M, Norman KE, Pépin A and Leroux A (1999). Walking after spinal cord injury: Evaluation, treatment, and functional recovery. Archives of Physical Medicine and Rehabilitation 80, 225–235. 10.1016/S0003-9993(99)90126-0 10025502

[r3] Carmo AA, Kleiner AF, Lobo da Costa PH and Barros RM (2012). Three-dimensional kinematic analysis of upper and lower limb motion during gait of post-stroke patients. Brazilian Journal of Medical and Biological Research 45, 537–545. URL: https://click.endnote.com/viewer?doi=10.1590%2Fs0100-879x2012007500051&token=WzIwMDA1MDcsIjEwLjE1OTAvczAxMDAtODc5eDIwMTIwMDc1MDAwNTEiXQ.dESJ3vAeIjIAlHzLL1hLNPiLKvk, 10.1590/S0100-879X2012007500051 22473324 PMC3854295

[r4] Chang HC, Hsu YL, Yang SC, Lin JC and Wu ZH (2016). A wearable inertial measurement system with complementary filter for gait analysis of patients with stroke or Parkinson’s disease. IEEE Access 4, 8442–8453. 10.1109/ACCESS.2016.2633304

[r5] Conforto S, D’Alessio T and Pignatelli S (1999). Optimal rejection of movement artefacts from myoelectric signals by means of a wavelet filtering procedure. Journal of Electromyography and Kinesiology 9, 47–57. 10.1016/S1050-6411(98)00023-6 10022561

[r6] Crowninshield RD and Brand RA (1981). A physiologically based criterion of muscle force prediction in locomotion. Journal of Biomechanics 14, 793–801. 10.1016/0021-9290(81)90035-X 7334039

[r7] Davy DT and Audu ML (1987). A dynamic optimization technique for predicting muscle forces in the swing phase of gait. Journal of Biomechanics 20, 187–201. 10.1016/0021-9290(87)90310-1 3571299

[r8] De Cock A, Vanrenterghem J, Willems T, Witvrouw E and De Clercq D (2008). The trajectory of the Centre of pressure during barefoot running as a potential measure for foot function. Gait & Posture 27, 669–675. 10.1016/J.GAITPOST.2007.08.013 17997096

[r9] Delp SL, Anderson FC, Arnold AS, Loan P, Habib A, John CT, Guendelman E and Thelen DG (2007). OpenSim: Open-source software to create and analyze dynamic simulations of movement. IEEE Transactions on Bio-medical Engineering 54, 1940–1950. 10.1109/TBME.2007.901024 18018689

[r10] Dickens WE and Smith MF (2006). Validation of a visual gait assessment scale for children with hemiplegic cerebral palsy. Gait & Posture 23, 78–82. 10.1016/J.GAITPOST.2004.12.002 16311198

[r11] Durandau G, Farina D and Sartori M (2018). Robust real-time musculoskeletal modeling driven by electromyograms. IEEE Transactions on Biomedical Engineering 65, 556–564. 10.1109/TBME.2017.2704085 28504931

[r12] Embrey DG, Holtz SL, Alon G, Brandsma BA and McCoy SW (2010). Functional electrical stimulation to dorsiflexors and plantar flexors during gait to improve walking in adults with chronic hemiplegia. Archives of Physical Medicine and Rehabilitation 91, 687–696. 10.1016/J.APMR.2009.12.024 20434604

[r13] Fukutoku K, Nozaki T and Murakami T (2020). Measurement of joint moments using wearable sensors. IEEJ Journal of Industry Applications 9, 125–131. 10.1541/ieejjia.9.125

[r14] Han J, Ding Q, Xiong A and Zhao X (2015). A state-space EMG model for the estimation of continuous joint movements. IEEE Transactions on Industrial Electronics 62, 4267–4275. 10.1109/TIE.2014.2387337

[r15] Heine R, Manal K and Buchanan TS (2011). Using hill-type muscle models and EMG data in a forward dynamic analysis of joint moment. 03, 169–186. 10.1142/S0219519403000727

[r16] Jamshidi N, Rostami M, Najarian S, Menhaj MB, Saadatnia M and Salami F (2010). Differences in center of pressure trajectory between normal and steppage gait. Journal of Research in Medical Sciences 15, 33. URL: https://www.ncbi.nlm.nih.gov/pmc/articles/PMC3082780/21526056 PMC3082780

[r17] Karatsidis A, Bellusci G, Schepers HM, de Zee M, Andersen MS and Veltink PH (2017). Estimation of Ground Reaction Forces and Moments During Gait Using Only Inertial Motion Capture. Sensors (Basel, Switzerland) 17. URL: https://www.ncbi.nlm.nih.gov/pmc/articles/PMC5298648/, 10.3390/S17010075 PMC5298648 28042857

[r18] Kesar TM, Perumal R, Reisman DS, Jancosko A, Rudolph KS, Higginson JS and Binder-Macleod SA (2009). Functional electrical stimulation of ankle plantarflexor and dorsiflexor muscles. Stroke 40, 3821–3827. 10.1161/STROKEAHA.109.560375 19834018 PMC2827197

[r19] Khurelbaatar T, Kim K, Lee SK and Kim YH (2015). Consistent accuracy in whole-body joint kinetics during gait using wearable inertial motion sensors and in-shoe pressure sensors. Gait & Posture 42, 65–69. 10.1016/J.GAITPOST.2015.04.007 25957652

[r20] Klöpfer-Krämer I, Brand A, Wackerle H, Müßig J, Kröger I and Augat P (2020). Gait analysis – Available platforms for outcome assessment. Injury 51, S90–S96. 10.1016/J.INJURY.2019.11.011 31767371

[r21] Knarr BA, Reisman DS, Binder-Macleod SA and Higginson JS (2014). Changes in predicted muscle coordination with subject-specific muscle parameters for individuals after stroke. Stroke Research and Treatment 2014. 10.1155/2014/321747 PMC4096388 25093141

[r22] Li G, Liu T, Yi J, Wang H, Li J and Inoue Y (2016). The lower limbs kinematics analysis by wearable sensor shoes. IEEE Sensors Journal 16, 2627–2638. 10.1109/JSEN.2016.2515101

[r23] Liu T, Inoue Y, Shibata K, Shiojima K and Han MM (2014). Triaxial joint moment estimation using a wearable three-dimensional gait analysis system. Measurement 47, 125–129. 10.1016/J.MEASUREMENT.2013.08.020

[r24] Manal K, Gravare-Silbernagel K and Buchanan TS (2012). A real-time EMG-driven musculoskeletal model of the ankle. Multibody system dynamics. 10.1007/s11044-011-9285-4 PMC3571695 23419878

[r25] Modenese L, Ceseracciu E, Reggiani M and Lloyd DG (2016). Estimation of musculotendon parameters for scaled and subject specific musculoskeletal models using an optimization technique. Journal of Biomechanics 49, 141–148. 10.1016/J.JBIOMECH.2015.11.006 26776930

[r26] Mohan DM, Khandoker AH, Wasti SA, Ismail Ibrahim Ismail Alali S, Jelinek HF and Khalaf K (2021). Assessment methods of post-stroke gait: A scoping review of technology-driven approaches to gait characterization and analysis. Frontiers in Neurology 12, 650024. 10.3389/FNEUR.2021.650024/BIBTEX 34168608 PMC8217618

[r27] Nadeau S, Betschart M and Béthoux F (2013). Article in physical medicine and rehabilitation clinics of North America. Physical Medicine and Rehabilitation Clinics of North America 24, 265–276. 10.1016/j.pmr.2012.11.007 23598262

[r28] Noamani A, Nazarahari M, Lewicke J, Vette AH and Rouhani H (2020). Validity of using wearable inertial sensors for assessing the dynamics of standing balance. Medical Engineering & Physics 77, 53–59. 10.1016/J.MEDENGPHY.2019.10.018 31926830

[r29] Patterson M and Caulfield B (2011). A novel approach for assessing gait using foot mounted accelerometers. In 2011 5th International Conference on Pervasive Computing Technologies for Healthcare and Workshops, PervasiveHealth 2011, pp. 218–221. IEEE https://eudl.eu/doi/10.4108/icst.pervasivehealth.2011.246061

[r30] Pedotti A, Krishnan VV and Stark L (1978). Optimization of muscle-force sequencing in human locomotion. Mathematical Biosciences 38, 57–76. 10.1016/0025-5564(78)90018-4

[r31] Peterson CL, Hall AL, Kautz SA and Neptune RR (2010). Pre-swing deficits in forward propulsion, swing initiation and power generation by individual muscles during hemiparetic walking. Journal of Biomechanics 43, 2348–2355. 10.1016/J.JBIOMECH.2010.04.027 20466377 PMC2922425

[r32] Ren L, Jones RK and Howard D (2008). Whole body inverse dynamics over a complete gait cycle based only on measured kinematics. Journal of Biomechanics 41, 2750–2759. 10.1016/J.JBIOMECH.2008.06.001 18672243

[r33] Revi DA, Alvarez AM, Walsh CJ, De Rossi SM and Awad LN (2020). Indirect measurement of anterior-posterior ground reaction forces using a minimal set of wearable inertial sensors: From healthy to hemiparetic walking. Journal of Neuroengineering and Rehabilitation 17, 1–13. URL: https://jneuroengrehab.biomedcentral.com/articles/10.1186/s12984-020-00700-7; http://creativecommons.org/publicdomain/zero/1.0/, 10.1186/S12984-020-00700-7/FIGURES/5 32600348 PMC7322880

[r34] Rueterbories J, Spaich EG and Andersen OK (2013). Characterization of gait pattern by 3D angular accelerations in hemiparetic and healthy gait. Gait & Posture 37, 183–189. 10.1016/J.GAITPOST.2012.06.029 22840891

[r35] Sartori M, Reggiani M, Farina D and Lloyd DG (2012). EMG-driven forward-dynamic estimation of muscle force and joint moment about multiple degrees of freedom in the human lower extremity. PLoS One 7, e52618. 10.1371/journal.pone.0052618 23300725 PMC3530468

[r36] Schutte LM, Rodgers MM, Zajac FE and Glaser RM (1993). Improving the efficacy of electrical stimulation-induced leg cycle ergometry: An analysis based on a dynamic musculoskeletal model. IEEE Transactions on Rehabilitation Engineering 1, 109–125. 10.1109/86.242425

[r37] Simonetti D, Hendriks M, Herijgers J, Cuerdo del Rio C, Koopman B, Keijsers N and Sartori M (2023). Automated spatial localization of ankle muscle sites and model-based estimation of joint torque post-stroke via a wearable sensorised leg garment. Journal of Electromyography and Kinesiology 72, 102808. URL: https://linkinghub.elsevier.com/retrieve/pii/S1050641123000676, 10.1016/J.JELEKIN.2023.102808 37573851

[r38] Simpson CS, Sohn MH, Allen JL and Ting LH (2015). Feasible muscle activation ranges based on inverse dynamics analyses of human walking. Journal of Biomechanics 48, 2990–2997. 10.1016/J.JBIOMECH.2015.07.037 26300401 PMC4592831

[r39] Stetter BJ, Krafft FC, Ringhof S, Stein T and Sell S (2020). A machine learning and wearable sensor based approach to estimate external knee flexion and adduction moments during various locomotion tasks. Frontiers in Bioengineering and Biotechnology 8, 508120. 10.3389/FBIOE.2020.00009/BIBTEX PMC6993119 32039192

[r40] Tagliapietra L, Vivian M, Sartori M, Farina D and Reggiani M (2015). Estimating EMG signals to drive neuromusculoskeletal models in cyclic rehabilitation movements. In Proceedings of the Annual International Conference of the IEEE Engineering in Medicine and Biology Society, EMBS. Institute of Electrical and Electronics Engineers Inc., pp. 3611–3614. 10.1109/EMBC.2015.7319174 26737074

[r41] Titianova EB and Tarkka IM (1995). Asymmetry in walking performance and postural sway in patients with chronic unilateral cerebral infarction. Journal of Rehabilitation Research and Development 32, 236–244 8592295

[r42] Wang H, Basu A, Durandau G and Sartori M (2022). Comprehensive kinetic and EMG dataset of daily locomotion with 6 types of sensors. URL: https://zenodo.org/record/6457662, 10.5281/ZENODO.6457662

[r43] Weygers I, Kok M, Konings M, Hallez H, De Vroey H and Claeys K (2020). Inertial sensor-based lower limb joint kinematics: A methodological systematic review. Sensors 20, 673. URL: https://www.mdpi.com/1424-8220/20/3/673/htm; https://www.mdpi.com/1424-8220/20/3/673, 10.3390/S20030673. 31991862 PMC7038336

[r44] Winter DA (2009). Biomechanics and Motor Control of Human Movement. John Wiley \& Sons. URL: https://books.google.nl/books?hl=it&lr=&id=_bFHL08IWfwC&oi=fnd&pg=PR13&dq=david+winter+biomechanics&ots=JnjAdtfgO5&sig=apv__aakj4SaRgxyHYcr0bdVT9w&redir_esc=y#v=onepage&q=davidwinterbiomechanics&f=false

[r45] Wu W, Saul KR and Huang H (2021). Using reinforcement learning to estimate human joint moments from electromyography or joint kinematics: An alternative solution to musculoskeletal-based biomechanics. Journal of Biomechanical Engineering 143. 10.1115/1.4049333/1092397 33332536

