# Supplementary material for: A wearable gait lab powered by sensor-driven digital twins for quantitative biomechanical analysis post-stroke
Source: Wearable Technol. 2024 Nov 14;5:e13. doi: 10.1017/wtc.2024.14 (PMC11579882; doi:10.1017/wtc.2024.14)
Supplement: Simonetti et al. supplementary material [file S2631717624000148sup001.pdf]

1. Supplementary Material

1.1. Experimental procedures

We used an open source dataset we previously created [2] with nine healthy participants (age = 25.4 ± 2.7 years, height = 173 ± 8 cm, weight: 68.1 ± 9.0 kg) performing walking trials at three different speeds (speed: 0.9, 1.8, 2.7 km/h). The participants were instrumented with nine bipolar EMG sensors on the dominant leg. For the purpose of the current work, we focused exclusively on the EMGs covering the muscles spanning the ankle joint (soleus, medial gastrocnemius, lateral gastrocnemius, tibialis anterior). Whole-body Kinematics were recorded using five IMUs (Xsens Link, Enschede, The Netherlands) and an infrared light camera motion tracking system (6+ series, Qualisys, Gothenburg, Sweden). The IMUs were placed on the dominant leg (thigh, tibia, and foot), one on the contralateral foot, and another on the pelvis. Ground reaction force (GRF) data were collected via a split-belt instrumented treadmill (Motek-Forcelink B.V, Culemborg, The Netherlands). A more detailed set-up and experimental procedure can be found in [3].

1.1.1. Data processing

For the healthy participants, the available normalized activation envelopes [2], obtained by processing the EMGs following the steps described by [1], were directly used as normalized muscle-specific linear envelopes.

2. Results

Figure 1 shows the comparison between reference and IMU-based knee and ankle angles (Figure 1.a), 3D GRFs (Figure 1.b) and ID ankle dorsi-plantar flexion torques (Figure 1.c) for each walking speed averaged across all gait cycles and all healthy participants. Reference and IMU-based joint angles, vertical GRFs show a similar shape and overlapping standard deviations, while major shape differences are noticeable between anterior-posterior and mediolateral GRFs, and ankle dorsi-plantar flexion torques.

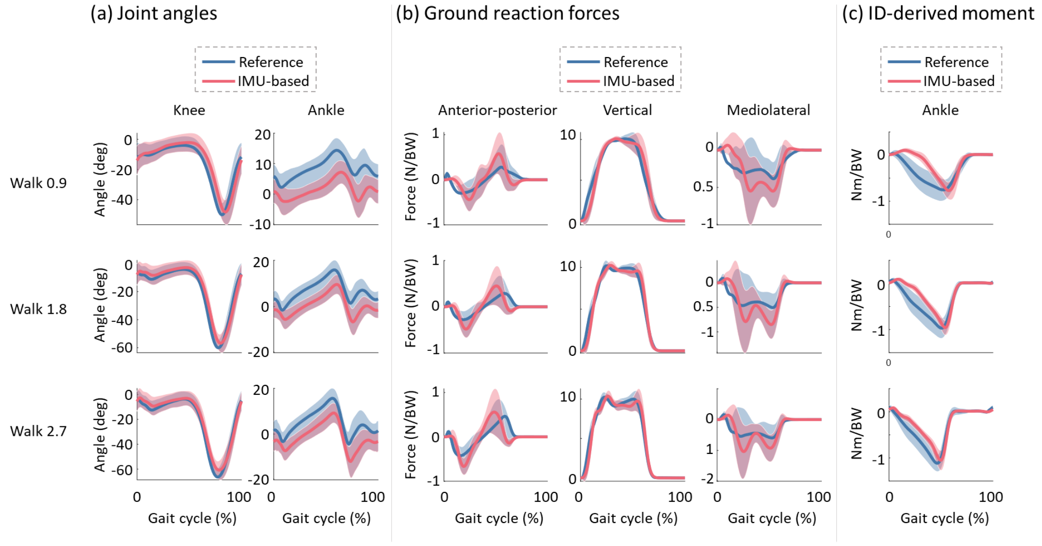

Figure 1. *Comparison of reference (in blue) and estimated (in red) joint angles (a), 3D GRFs (b) and inverse dynamics derived ankle dorsi-plantar flexion torques (c) averaged across all gait cycles and all healthy participants for each walking speed (walk 0.9 km/h, walk 1.8 km/h, walk 2.7 km/h). The solid lines represent the mean values while the shaded area is the standard deviation.*

Figure 2 shows the comparison between reference and IMU-based CoP trajectory in the anterior-posterior and mediolateral directions. For all speeds, the IMU-based CoP trajectory in the anterior-posterior directions showed a similar trend and overlapping standard deviations with respect to the reference CoP trajectory. A marked difference is visible for the CoP trajectory in the mediolateral direction.

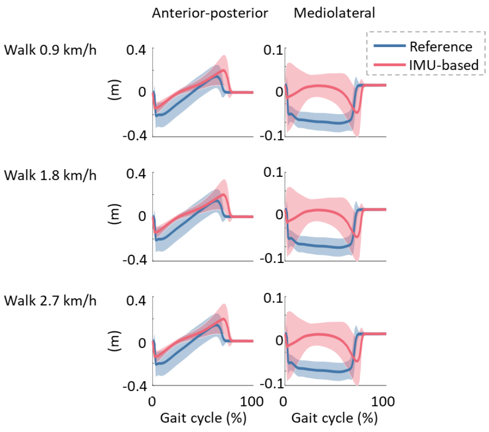

Figure 2. *Comparison of reference (in blue) and estimated (in red) center of pressure (CoP) averaged across all gait cycles and all healthy participants for each walking speed (walk 0.9 km/h, walk 1.8 km/h, walk 2.7 km/h). The solid lines represent the mean values while the shaded area is the standard deviation.*

Table 1 shows the R^2 and RMSE between reference and IMU-based kinematics and kinetic data for healthy participants. For the knee angles, R^2 and RMSE averaged across all participants and all walking speeds ranged between 0.6 and 0.99 with a mean (± std) of 0.92 ± 0.08, and between 3.2 deg and 11.8 deg with a mean (± std) of 5.9 ± 2.67 deg, respectively. For the ankle angles, R^2 and RMSE averaged across all participants and all walking speeds ranged between 0.13 and 0.96 with a mean (± std) of 0.73 ± 0.18, and between 3.2 deg and 11.8 deg with a mean (± std) of 6.9 ± 2.72 deg, respectively. More details are presented in Table 1.

Table 1. *R^2 and RMSE values between experimental and estimated flexion-extension angle of knee and angle, 3D GRFs, 3D CoP and ID-derived ankle dorsi-plantar flexion torques during walking at three different speeds for healthy participants.*

Speed	Joint angles		GRFs			CoP		ID-torque
(km/h)	Knee	Ankle	AP*	V*	ML*	AP*	ML*	Ankle
walk 0.9	0.88 ± 0.05	0.62 ± 0.05	0.40 ± 0.04	0.91 ± 0.03	0.48 ± 0.05	0.24 ± 0.21	0.08 ± 0.11	0.55 ± 0.04
walk 1.9	0.94 ± 0.03	0.77 ± 0.05	0.45 ± 0.02	0.96 ± 0.03	0.59 ± 0.03	0.40 ± 0.21	0.11 ± 0.16	0.78 ± 0.05
walk 2.7	0.94 ± 0.10	0.81 ± 0.08	0.40 ± 0.02	0.95 ± 0.05	0.55 ± 0.04	0.48 ± 0.18	0.15 ± 0.21	0.82 ± 0.06
Speed	Knee	Ankle	AP*	V*	ML*	AP*	ML*	Ankle
(km/h)	(deg)	(deg)	(N/kg)	(N/kg)	(N/kg)	(m)	(m)	(Nm/kg)
walk 0.9	6.32 ± 0.94	7.00 ± 0.27	0.26 ± 0.01	1.34 ± 0.18	0.22 ± 0.01	0.14 ± 0.07	0.09 ± 0.03	0.28 ± 0.02
walk 1.8	5.61 ± 0.55	6.79 ± 0.08	0.44 ± 0.01	0.91 ± 0.16	0.33 ± 0.01	0.12 ± 0.06	0.08 ± 0.03	0.22 ± 0.03
walk 2.7	5.70 ± 2.05	6.77 ± 0.35	0.57 ± 0.01	0.95 ± 0.27	0.41 ± 0.01	0.12 ± 0.05	0.08 ± 0.03	0.22 ± 0.03

* AP = anterior-posterior; V = vertical; ML = mediolateral

For the GRFs, R^2 and RMSE averaged across all healthy participants and for all walking speeds were 0.42 ± 0.15 and 0.43 ± 0.16 N/kg in the anterior-posterior direction, 0.94 ± 0.04 and 1.07 ± 0.31 N/kg in the vertical direction, and 0.54 ± 0.23 and 0.33 ± 0.14 N/kg in the mediolateral direction, respectively. For the CoP trajectory, R^2 and RMSE averaged across all healthy participants and for all walking speeds were 0.37 ± 0.23 and 0.05 ± 0.03 min the anterior-posterior direction, and 0.11 ± 0.17 and 0.13 ± 0.06 min the mediolateral direction, respectively. For the ID ankle dorsi-plantar flexion torques, R^2 and RMSE averaged across all healthy participants, and all walking speeds were 0.49 ± 0.24 and 0.32 ± 0.14 Nm/kg, respectively.

Figure 3 compares reference ID ankle dorsi-plantar flexion torque with Ref-EMG-driven and IMU-EMG-driven ankle dorsi-plantar flexion torques for each walking speed averaged across all gait cycles and healthy participants. The IMU-EMG-driven ankle dorsi-plantar flexion torques showed a better match of the negative plantar flexion peak with the reference ID-derived ankle dorsi-plantar flexion torques with respect to the Ref-EMG-driven ankle dorsi-plantar flexion torque. Both Ref-EMG-driven and IMU-EMG-driven ankle dorsi-plantar flexion torques, for all walking speeds, showed an earlier negative plantar flexion peak with respect to the reference ID ankle dorsi-plantar flexion torque.

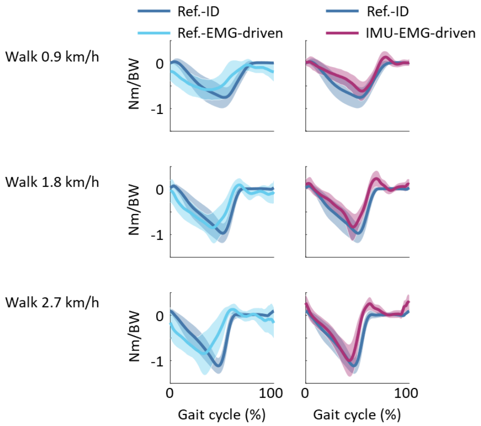

Figure 3. *Comparison between reference- and EMG-driven estimated ankle dorsi-plantar flexion torques. Across all participants and gait cycles and for each walking speed (walk 0.9 km/h, walk 1.8 km/h, walk 2.7 km/h), reference ankle torque (Ref-ID, dark blue line) is compared with (a) laboratory-derived signals- and EMG-driven torque (Ref-EMG-driven, light blue line), and IMU-based signal- and EMG-driven torque (IMU-EMG-driven, purple line).*

Table 2 shows the R^2 and RMSE between reference ID and both EMG-driven model output ankle dorsi-plantar flexion torques, *i.e.*, Ref-EMG-driven and IMU-EMG-driven, across all healthy participants and for all walking speeds. R^2 and RMSE averaged across all participants and speeds were 0.72 ± 0.16 and 0.24 ± 0.01 Nm/kg between reference ID and Ref-EMG-driven ankle dorsi-plantar flexion torques, and 0.72 ± 0.09 and 0.27% ± 0.11% between reference ID and IMU-EMG-driven ankle dorsi-plantar flexion torques, respectively.

References

[1] Konrad, P., 2005. The ABC of EMG A Practical Introduction to Kinesiological Electromyography URL: www.noraxon.com.

Table 2. *R^2 and normalized (by body weight) RMSE values between reference (Ref-ID) and both Ref-EMG-driven and IMU-EMG-driven ankle dorsi-plantar flexion torques across all healthy participants walking at three different speeds.*

R^2	Ref-EMG-driven	IMU-EMG-driven
walk 0.9 km/h	0.47 ± 0.08	0.68 ± 0.05
walk 1.8 km/h	0.56 ± 0.05	0.74 ± 0.03
walk 2.7 km/h	0.43 ± 0.05	0.73 ± 0.07
RMSE (Nm/kg)	Ref-EMG-driven	IMU-EMG-driven
walk 0.9 km/h	0.26 ± 0.03	0.25 ± 0.01
walk 1.8 km/h	0.30 ± 0.03	0.27 ± 0.02
walk 2.7 km/h	0.40 ± 0.05	0.31 ± 0.03

[2] Wang, H., Basu, A., Durandau, G., Sartori, M., 2022. Comprehensive Kinetic and EMG Dataset of Daily Locomotion with 6 types of Sensors URL: https://zenodo.org/record/6457662, doi: http://dx.doi.org/10.5281/ZENODO.6457662.

[3] Wang, H., Basu, A., Durandau, G., Sartori, M., 2023. A wearable real-time kinetic measurement sensor setup for human locomotion. Wearable Technologies 4, e11. URL: https://www.cambridge.org/core/journals/wearable-technologies/article/wearable-realtime-kinetic-measurement-sensor-setup-for-human-locomotion/488C21B7706FFDFA7FFAB387FD0A1A64, doi: http://dx.doi.org/10.1017/WTC.2023.7.
